# Supplementary figures and images for: Impact of Androgen Receptor Activity on Prostate-Specific Membrane Antigen Expression in Prostate Cancer Cells
Source: Int J Mol Sci. 2022 Jan 18;23(3):1046. doi: 10.3390/ijms23031046 (PMC8835452; doi:10.3390/ijms23031046)

Figure S1: ARB Motif Distribution

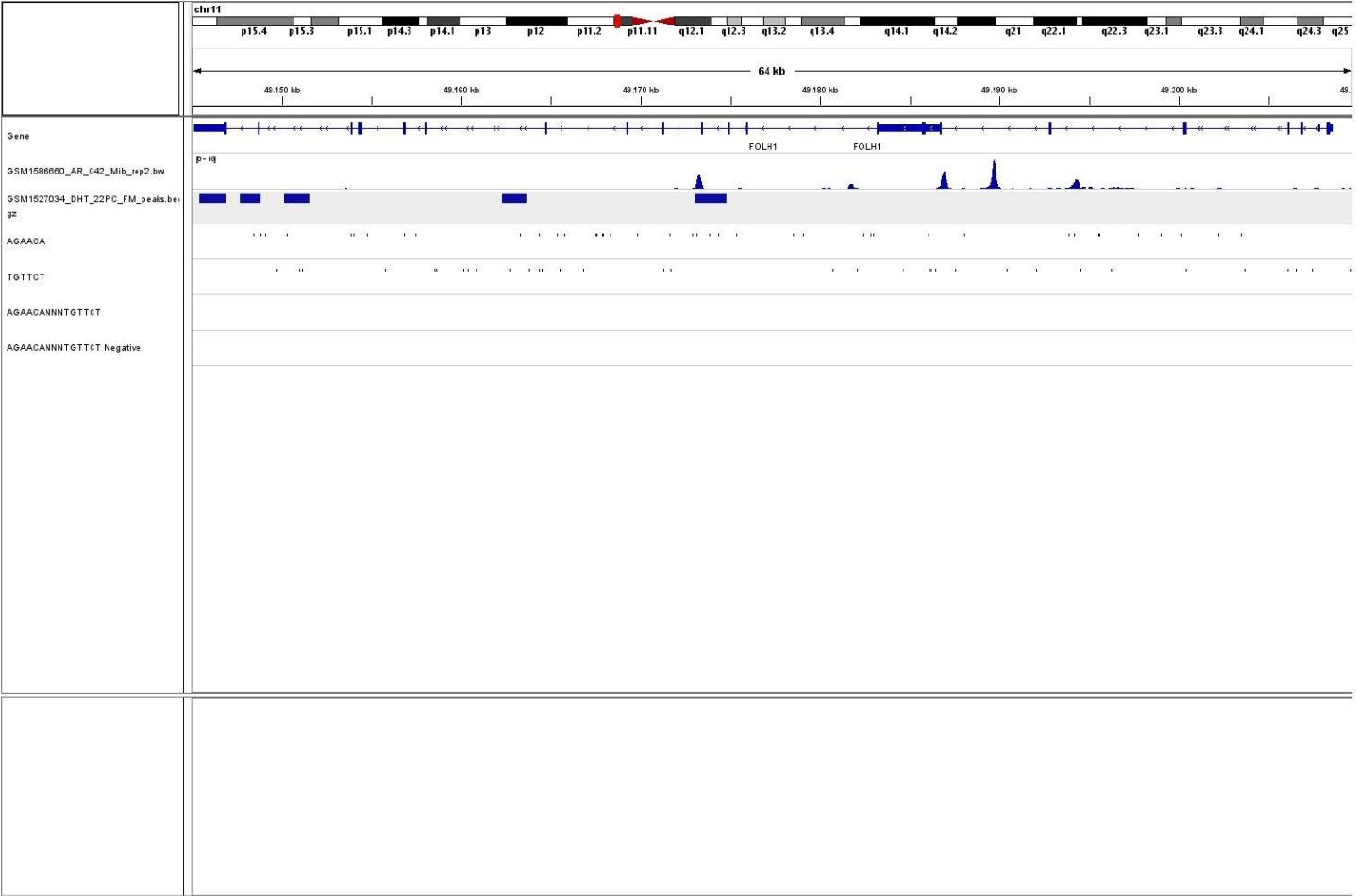

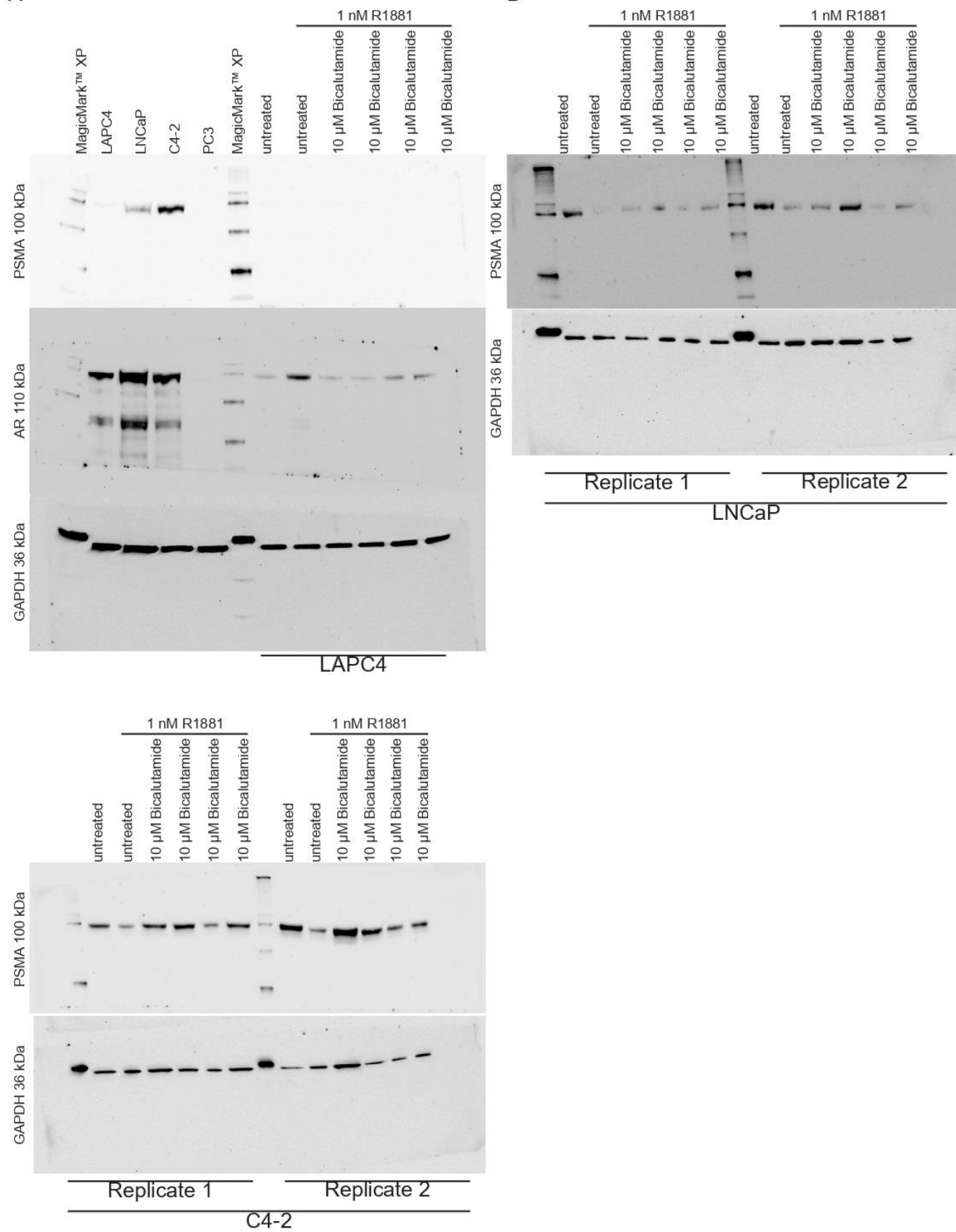

Supplement: Supplementary file 1 [file ijms-23-01046-s001.zip › ijms-1473502-SI.pdf]
